# Tofacitinib in chronic pouchitis: A step towards addressing an unmet need

**DOI:** 10.1002/ueg2.12691

**Published:** 2024-11-07

**Authors:** Jacob E. Ollech

**Affiliations:** ^1^ Division of Gastroenterology Faculty of Medicine, Rabin Medical Center, Tel‐Aviv University Tel Aviv Israel

Chronic pouchitis remains a significant challenge in the management of patients with ulcerative colitis (UC) who have undergone ileal pouch‐anal anastomosis.^1^ Despite advances in medical therapy, some patients continue to experience persistent symptoms, requiring novel therapeutic approaches. The recent pilot study by de Jong et al. “Tofacitinib for the treatment of chronic pouchitis; a pilot study”^2^ evaluating the efficacy of tofacitinib, a Janus kinase (JAK) inhibitor, represents an important step in addressing this unmet need.

Current treatment options for pouchitis, including antibiotics, immunomodulators, and biologics, may fail to provide sustained remission, leaving a proportion of patients with ongoing disease activity.^3^ The EARNEST trial was the first appropriately powered prospective randomized controlled trial evaluating the efficacy of an advanced therapy (vedolizumab) in chronic pouchitis.^4^ And while there are trials assessing other advanced therapies, almost all are retrospective. Indeed, beyond vedolizumab, there is a lack of robust prospective clinical trials of advanced therapies, making it challenging to establish evidence‐based treatment protocols for patients with refractory chronic pouchitis.

Tofacitinib is approved for treating moderately to severely active UC and has shown promise in case reports and retrospective series for treating chronic pouchitis.^5^, ^6^, ^7^ The study by de Jong et al. is the first prospective trial evaluating the efficacy and safety of tofacitinib in this challenging patient population.

The major strength of this study is its prospective design. By following patients systematically over time, the researchers collected detailed data on clinical, biochemical, endoscopic, and histologic outcomes. The granular data collected allows for a more nuanced understanding of the drug's impact on various aspects of disease activity.

The study's primary endpoint—clinical remission defined by a Pouchitis Disease Activity Index score of less than 7 and a reduction of at least 3 points from baseline—was achieved in 31% of patients after 8 weeks of treatment. Additionally, 54% of patients experienced a clinical response, indicating that tofacitinib can effectively reduce symptoms in many patients with chronic pouchitis.

The use of objective markers such as endoscopy and biomarkers is another notable strength of this study. In classic inflammatory bowel disease management, including Crohn's disease and UC, “treating to a target” using objective markers has been associated with better long‐term outcomes. A recently published endoscopic sub‐study of the EARNEST trial showed that patients with pouchitis achieving endoscopic remission were more likely to have better outcomes.^8^ Based on this, the concept of “treat to target” should probably be applied to chronic pouchitis as well. Moreover, the authors showed that calprotectin is a reliable surrogate marker of endoscopic inflammation in pouch patients, supporting its use in the monitoring and management of pouchitis, supporting previously reported studies.^9^, ^10^ These findings also add to the data supporting the use of objective marks of disease control in pouch patients.

Despite its strengths, the study is not without limitations. The small sample size of just 13 patients limits the generalizability of the findings. The study's open‐label design also introduces bias, as both patients and investigators were aware of the treatment being administered. Most importantly, the absence of a control group further complicates the interpretation of the results, as it is unclear how much of the observed improvement was due to the effect of the medication.

Despite these limitations, the pilot study by de Jong et al. offers promising preliminary evidence that tofacitinib may be a valuable addition to the therapeutic arsenal for chronic pouchitis. Some of the data reported also supports the use of objective markers of inflammation, including calprotectin, when monitoring patients with a pouch. Despite the limitations of a small sample size and short follow‐up, the study provides a foundation for further research into using JAK inhibitors in this challenging condition.

Future studies should aim to build on these findings by including larger patient populations, employing randomized controlled designs, and exploring longer treatment durations.

In conclusion, while much work remains to be done, this study represents a step forward in the quest to improve the management of chronic pouchitis.

## Data Availability

Data sharing is not applicable to this article as no new data were created or analyzed in this study.
